# Chitosan-Graft-Branched Polyethylenimine Copolymers: Influence of Degree of Grafting on Transfection Behavior

**DOI:** 10.1371/journal.pone.0034711

**Published:** 2012-04-11

**Authors:** Daniele Pezzoli, Francesca Olimpieri, Chiara Malloggi, Sabrina Bertini, Alessandro Volonterio, Gabriele Candiani

**Affiliations:** 1 Unità Politecnico di Milano, Consorzio Interuniversitario Nazionale per la Scienza e Tecnologia dei Materiali - INSTM, Milan, Italy; 2 Department of Chemistry, Materials and Chemical Engineering “Giulio Natta”, Politecnico di Milano, Milan, Italy; 3 Istituto di Ricerche Chimiche e Biochimiche G. Ronzoni, Milan, Italy; Aristotle University of Thessaloniki, Greece

## Abstract

**Background:**

Successful non-viral gene delivery currently requires compromises to achieve useful transfection levels while minimizing toxicity. Despite high molecular weight (MW) branched polyethylenimine (bPEI) is considered the gold standard polymeric transfectant, it suffers from high cytotoxicity. Inversely, its low MW counterpart is less toxic and effective in transfection. Moreover, chitosan is a highly biocompatible and biodegradable polymer but characterized by very low transfection efficiency. In this scenario, a straightforward approach widely exploited to develop effective transfectants relies on the synthesis of chitosan-*graft*-low MW bPEIs (Chi-*g*-bPEI_x_) but, despite the vast amount of work that has been done in developing promising polymeric assemblies, the possible influence of the degree of grafting on the overall behavior of copolymers for gene delivery has been largely overlooked.

**Methodology/Principal Findings:**

With the aim of providing a comprehensive evaluation of the pivotal role of the degree of grafting in modulating the overall transfection effectiveness of copolymeric vectors, we have synthesized seven Chi-*g*-bPEI_x_ derivatives with a variable amount of bPEI grafts (minimum: 0.6%; maximum: 8.8%). Along the Chi-*g*-bPEI_x_ series, the higher the degree of grafting, the greater the ζ-potential and the cytotoxicity of the resulting polyplexes. Most important, in all cell lines tested the intermediate degree of grafting of 2.7% conferred low cytotoxicity and higher transfection efficiency compared to other Chi-*g*-bPEI_x_ copolymers. We emphasize that, in transfection experiments carried out in primary articular chondrocytes, Chi-*g*-bPEI_2.7%_ was as effective as and less cytotoxic than the gold standard 25 kDa bPEI.

**Conclusions/Significance:**

This work underlines for the first time the pivotal role of the degree of grafting in modulating the overall transfection effectiveness of Chi-*g*-bPEI_x_ copolymers. Crucially, we have demonstrated that, along the copolymer series, the fine tuning of the degree of grafting directly affected the overall charge of polyplexes and, altogether, had a direct effect on cytotoxicity.

## Introduction

With the advent of DNA-based gene therapy as well as the discovery of short interfering (si)RNAs as the key mediators of RNA interference (RNAi), the development of delivery platforms for the systemic application of nucleic acids has gained particular relevance for the establishment of novel therapeutic strategies [1], [2]. Cationic polymers display striking advantages as vectors for gene delivery: they can be specifically tailored for the proposed application by selecting an appropriate molecular weight (MW) and/or coupling them to cell or tissue specific targeting moieties [3]. Nonetheless, their use in therapy is hampered by their still low transfection efficiency and high toxicity.

Since its introduction in 1995, polyethylenimine (PEI) has been considered the gold standard polymeric gene delivery vector [4]. PEI exists in both linear (lPEI) and branched (bPEI) form. It is worth noting that, although conflicting results are reported in literature, several authors have pointed out the superior transfection behavior of bPEI *in vitro* [5]. Moreover, Godbey showed a direct correlation between transfection efficiency and MW of bPEI used in transfection experiments (70 kDa bPEI>10 kDa bPEI>1.8 kDa bPEI) [6]. On the other hand, Kunath and coworkers demonstrated that low MW (LMW) PEI was less cytotoxic than its high MW (HMW) counterpart [7]. Efficacy and adverse reactions seem thereby to be strongly related and the successful non-viral gene delivery currently requires compromises to achieve a useful level of transfection efficiency while minimizing the toxicity [8].

Chitosan, a naturally-derived linear aminopolysaccharide obtained by the deacetylation of chitin, is composed of a random distribution of D-glucosamine (GlcN), with a primary amino group that confers high positive charge at acidic or neutral pH, and *N*-acetyl-D-glucosamine (GlcNAc) linked by *β*(1→4) glycosidic bonds [9]. Chitosan is a highly biocompatible and biodegradable gene delivery vector [10]. However, its cellular uptake is limited, as for other non-derivatized aminoglycosides [11], and it still suffers from lower transfection efficiency compared to PEI [12].

In this scenario, a straightforward approach to overcome the drawbacks of both PEI and chitosan is based on the synthesis of copolymer derivatives. Indeed, different chitosan-*graft*-bPEIs (Chi-*g*-bPEIs) have shown lower cytotoxicity and enhanced transfection efficiency compared to the highly effective HMW bPEI [13]–[15]. For instance, two independent studies by Jiang [13] and Wong [16] reported different strategies to synthesize Chi-*g*-bPEIs that relied on the imine formation between LMW bPEI grafts and periodate-oxidized chitosan and on the cationic polymerization of aziridine in presence of chitosan, respectively. In recent studies Chi-*g*-bPEI copolymers have been further functionalized with targeting moieties such as galactose, mannose, folate, and cyclodextrin, demonstrating superior efficiencies both *in vitro* and *in vivo* [17]–[20]. Altogether, these data disclose Chi-*g*-PEIs as promising tools for gene delivery applications.

Despite the vast amount of work that has been done in developing effective gene delivery vectors, to our knowledge, the possible influence of the degree of grafting on the overall behavior of copolymers intended for gene delivery applications has been largely overlooked. Indeed, amid theoretically infinite combinations of Chi-*g*-bPEIs that could have been synthesized, only few copolymers have been investigated and their comparative evaluation in terms of overall transfection effectiveness has never been adequately addressed before [13]–[16], [21], [22]. On these premises, we have synthesized a series of seven Chi-*g*-bPEI copolymers with different degrees of grafting of bPEI onto the chitosan backbone. The aim of our study was to investigate the role of the degree of grafting on the cytotoxicity and on the transfection efficiency of copolymer-based polyplexes. Furthermore, in order to provide for the first time valuable guidelines for the development of more effective copolymers for gene delivery, we performed structure-activity relationship studies to ascertain possible correlations bridging the chemical structure (degrees of PEI grafting) of copolymers to the physico-chemical properties of Chi-*g*-bPEI/DNA complexes and their biological outcome.

## Results and Discussion

### Synthesis and characterization of Chi-g-bPEI_x_ copolymers

**Figure 1:**
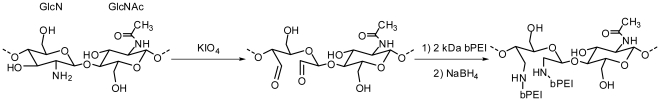
Synthesis of Chi-*g*-bPEI_x_ copolymer. General reaction scheme for the synthesis of Chi-*g*-bPEI_x_ copolymers by grafting 2 kDa branched polyethylenimine (bPEI) onto the oxidized D-glucosamine (GlcN) unit of chitosan. *N*-acetyl-D-glucosamine (GlcNAc) is also reported.

**Figure 2:**
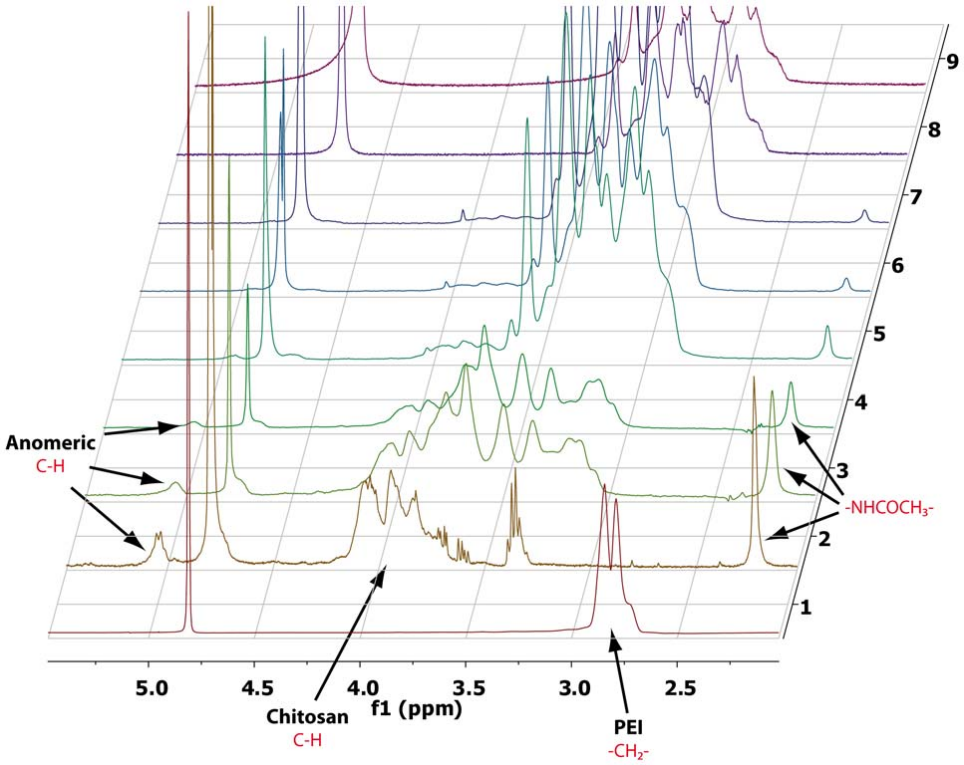
^1^H NMR spectra of bPEI, chitosan and Chi-*g*-bPEI_x_ copolymers. ^1^H NMR spectra of (1) 2 kDa branched polyethylenimine (bPEI), (2) oxidized chitosan, (3) Chi-*g*-bPEI_0.6%_, (4) Chi-*g*-bPEI_2.4%_, (5) Chi-*g*-bPEI_2.7%_, (6) Chi-*g*-bPEI_5.2%_, (7) Chi-*g*-bPEI_7.0%_, (8) Chi-*g*-bPEI_8.7%_ and (9) Chi-*g*-bPEI_8.8%_ copolymers. All spectra were recorded using D_2_O as solvent.

The fine tuning of the reaction conditions described by Jiang and colleagues [13]–[15] has allowed us to synthesize a series of seven Chi-*g*-bPEI_x_ copolymers with different degrees of grafting (Fig. 1), starting from commercially sourced medium MW (MMW) chitosan and LMW bPEI. Of note, although chitosan was insoluble in water under neutral conditions [23], Chi-*g*-bPEI_x_ derivatives were completely soluble at physiological pH because of the hydrophilic behavior of bPEI, as previously reported by others [13]. Amine quantification by TNBSA assay was used to quantify the amount of bPEI grafted onto the chitosan backbone. As expected, the degree of grafting of copolymers increased by increasing the amount of 2 kDa bPEI added to the periodate-oxidized chitosan during the synthesis (Table 1). However, beyond the addition of 6.35×10^−1^ equiv of bPEI per GlcN unit, the degree of grafting increased more slowly probably owing to the steric hindrance of bPEI and reached the maximum value of 8.8±1.3% after the addition of 6.35 equiv. The grafting of bPEI onto the oxidized chitosan was further assessed by ^1^H NMR analysis. Indeed, the spectra of Chi-*g*-bPEI_x_ showed the presence of either peaks resonating at 3.5-2.5 ppm belonging to bPEI and those of chitosan at 4.0-3.4 ppm (Fig. 2). Moreover, the relative intensity of the latter, the singlet of the acetyl groups of the GlcNAc moieties at 2.5- 2.0 ppm and the smaller signal of anomeric protons around 5.0 ppm clearly decreased by increasing the amount of bPEI grafted onto the oxidated chitosan (Fig. 2, spectra 3–9).

**Table 1:** Degree of grafting of Chi-*g*-bPEI_x_ copolymers as a function of 2 kDa bPEI equiv per D-glucosamine unit added during the synthesis.

Chi-*g*-bPEI_x_ copolymer	bPEI equiv of reaction	Degree of grafting (x)^a^
Chi-*g*-bPEI_0.6%_	1.27×10^−2^	0.6±0.1%
Chi-*g*-bPEI_2.4%_	6.35×10^−2^	2.4±0.1%
Chi-*g*-bPEI_2.7%_	1.27×10^−1^	2.7±0.1%
Chi-*g*-bPEI_5.2%_	6.35×10^−1^	5.2±0.3%
Chi-*g*-bPEI_7.0%_	2.54×10^0^	7.0±0.2%
Chi-*g*-bPEI_8.7%_	1.27×10^1^	8.7±0.1%
Chi-*g*-bPEI_8.8%_	6.35×10^0^	8.8±1.3%

a “x” is the average percentage of D-glucosamine (GlcN) monomers grafted with branched polyethylenimine (bPEI). Results are expressed as mean ± standard deviation.

### Complexation and condensation abilities of (co)polymers

**Figure 3:**
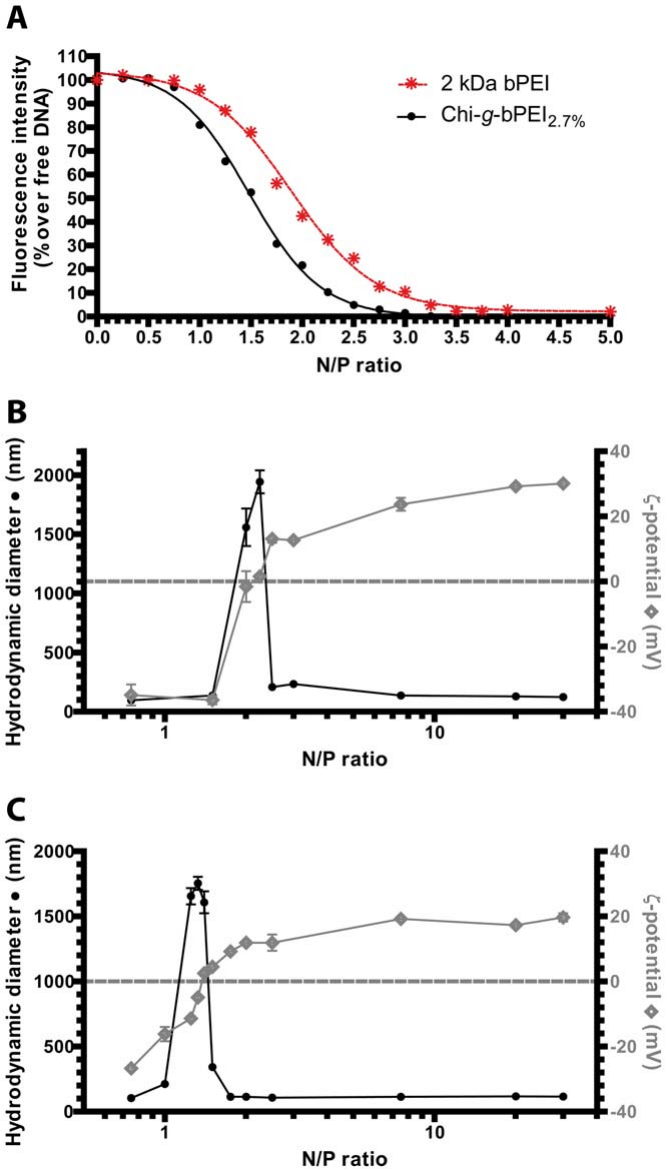
DNA complexation abilities of Chi-*g*-bPEI_2.7%_ and 2 kDa bPEI. Comparative DNA complexation ability of 2 kDa branched polyethylenimine (bPEI) (red stars and dotted line) and the model copolymer Chi-*g*-bPEI_2.7%_ (black cycles and solid line) evaluated by monitoring the fluorochrome exclusion from polyplexes as a function of nitrogen (N) to plasmid DNA phosphate (P) ratio (N/P). Average hydrodynamic diameters (full black circles) and ζ-potentials (empty grey rhombi) of (B) 2 kDa bPEI and (C) Chi-*g*-bPEI_2.7%_ determined over a wide range of N/P ratio. Results are expressed as mean ± standard deviation (n≥3).

Given that the DNA complexation and condensation are prerequisites for effective transfection, we have first evaluated by fluorescence-exclusion assay [24], [25] the DNA complexation behavior of Chi-*g*-bPEI_x_ and those of the starting materials, chitosan and 2 kDa bPEI as a function of the N/P ratio. SYBR Green I is a non-specific intercalating probe that gives a strong fluorescence signal when bound to DNA but only very weak fluorescence emission when it is free in solution [26]. Therefore, when the plasmid is completely complexed by a polycation, the fluorochrome cannot intercalate the DNA anymore and its fluorescence intensity dramatically decreases [27]. Interestingly, all the complexation curves of Chi-*g*-bPEI_x_ and chitosan superimposed almost perfectly (Fig. S1) and shared a sigmoidal decrease in fluorescence that reached a bottom plateau at nitrogen (N) to plasmid DNA phosphate (P) (N/P) ratios ≥2.75, as shown in Fig. 3A for the model copolymer Chi-*g*-bPEI_2.7%_. Inversely, unconjugated 2 kDa bPEI was less effective than Chi-*g*-bPEI_x_ copolymers in complexing DNA (bottom plateau at N/P≥3.5), probably because of its low MW [7]. In addition, gel retardation assay was carried out in order to further support the DNA binding ability of copolymers and the stability of the Chi-*g*-bPEI_x_/DNA complexes [13], [16]. Irrespective of the degree of grafting, Chi-*g*-bPEI_x_ were able to completely retain the DNA in the agarose gel slots at N/P≥3 (Fig. S2). Of note, despite differences in starting materials and/or linking strategies adopted, our results are consistent with the literature [13], [16]. A possible explanation for differences in complexation behavior between Chi-*g*-bPEI_x_ and LMW bPEI relies on the more extended conformation of the former, as previously suggested by Wong and colleagues [16] and on the overall too weak binding per molecule to the DNA of the latter [28], [29]. In parallel, we checked for possible differences in DNA condensation ability of 2 kDa bPEI and the model copolymer Chi-*g*-bPEI_2.7%_, by evaluating the size and the overall charge of the corresponding polyplexes over a wide range of N/P ratios (Fig. 3B and 3C, respectively). Typical ζ-potential curves had a marked sigmoid shape as a function of N/P ratio, with the inversion point (0 mV) corresponding to a spike in size distribution profiles due to polyplex aggregation [30], [31]. In agreement with the aforementioned results shown in Fig. 3A, Chi-*g*-bPEI_2.7%_ was also more efficient in packing nucleic acids than unconjugated bPEI as evidenced by the displacement of its size and ζ-potential curves to lower N/P ratios.

### Biological and physico-chemical characterization of polyplexes

**Figure 4:**
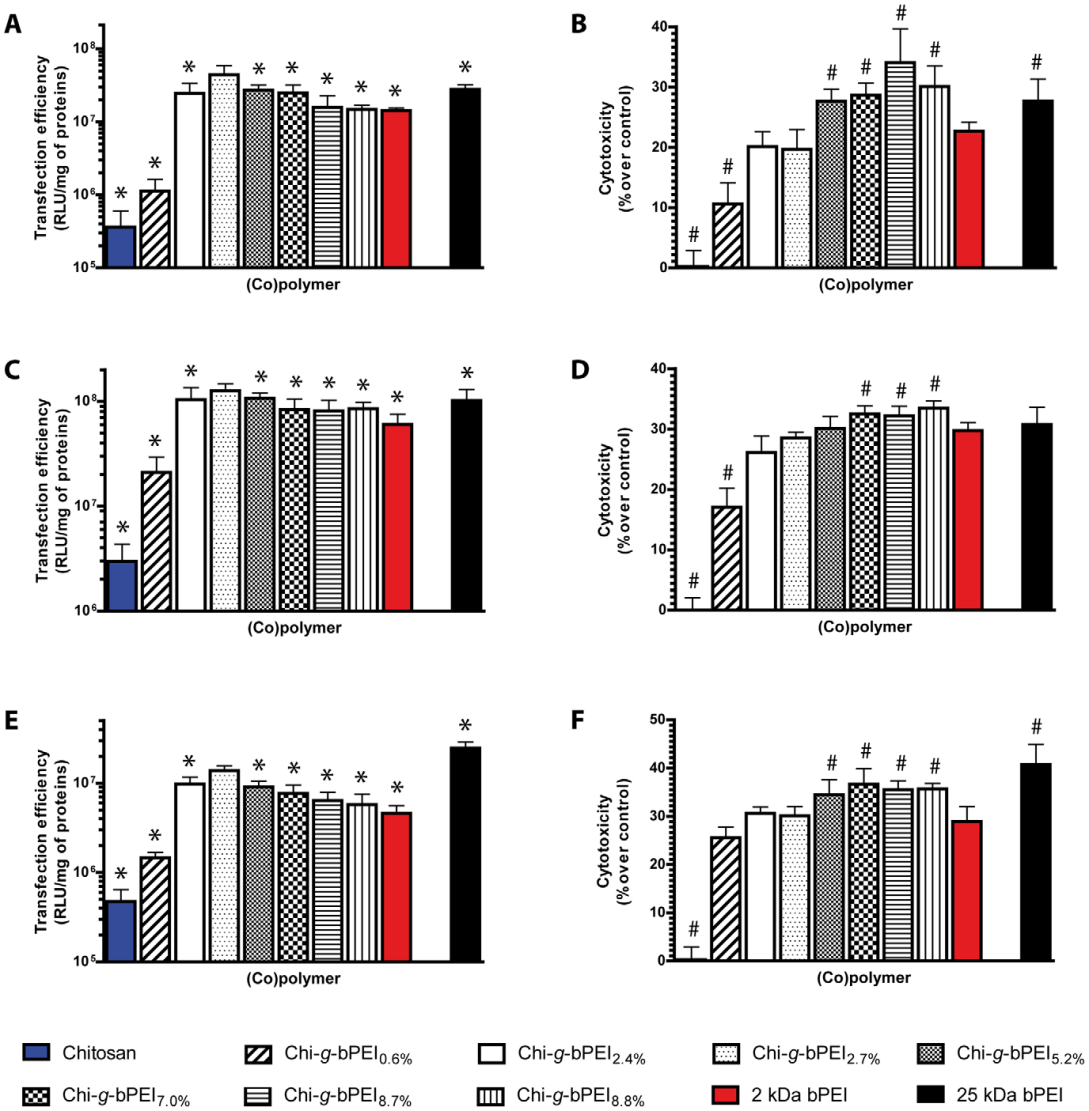
Transfection efficiencies and cytotoxicities of chitosan, Chi-*g*-bPEI_x_ and bPEIs in three cell lines. Transfection efficiencies (A, C, E) and cytotoxicities (B, D, F) in HeLa (A, B), COS-7 (C, D) and U87-MG (E, F) cell lines. Chitosan, Chi-*g*-bPEI_x_ copolymers and 2 kDa branched polyethylenimine (bPEI) were used at nitrogen (N) to plasmid DNA phosphate (P) ratio (N/P) 30 and 25 kDa bPEI was administered at N/P 10 according to the existing literature. Results are expressed as mean ± standard deviation (n≥4) (*, *p*<0.05 *vs.* Chi-*g*-bPEI_2.7%_; #, *p*<0.05 *vs.* 2 kDa bPEI).

Since DNA complexation and condensation behaviors are not predictive of the transfection effectiveness of a gene delivery vector [32] and the use of serum cannot be avoided in long-term cell cultures, we tested the overall transfection behavior of Chi-*g*-bPEI_x_ copolymers as a function of N/P ratio in complete medium (DMEM with 10% Fetal Bovine Serum, FBS). Although this experimental condition is far from the *in vivo* situation, transfections carried out in serum-enriched medium are commonly used to check the serum resistance of gene delivery vectors prior to performing animal studies [33]. As expected, transfection efficiency increased with increasing N/P ratios (Fig. S3). It is worth noting that although DNA packing was effective at N/P≥2.75, transfection efficiencies of all copolymers were negligible for N/P≤5 in HeLa cells (not shown). At N/P 10 and irrespective of the degree of grafting, all Chi-*g*-bPEI_x_ except the less grafted Chi-*g*-bPEI_0.6%_ exhibited an average 4.7-fold increase in transfection efficiency compared to the unconjugated 2 kDa bPEI (*p*<0.05) (Fig. S3). Moreover, at higher N/P ratios, the transfection behavior of the Chi-*g*-bPEI_x_ series followed a bell-like trend with a well-defined narrow maximum represented by Chi-*g*-bPEI_2.7%_ (*p*<0.05 *vs.* all at N/P 20 and 30). Thus, the increase in bPEI grafts along the copolymer series yielded enhanced transfection for Chi-*g*-bPEI_2.7%_ at N/P 30, with transfection efficiencies up to 3.1- and 1.6-fold higher with respect to those of the unconjugated 2 kDa bPEI and the gold standard 25 kDa bPEI, respectively (Fig. 4A) (*p*<0.05). Although the precise reason behind such different behaviors is still an open question, beyond this optimum, the higher the degree of grafting, the lower the transfection effectiveness that, in turn, reached its lowest value for the most grafted Chi-*g*-bPEI_8.8%_ (transfection efficiency: Chi-*g*-bPEI_8.8%_ *vs.* 2 kDa bPEI, not statistically significant). Interestingly, there was a direct correlation between the degree of grafting of Chi-*g*-bPEI_x_ copolymers and their cytotoxicities in HeLa cell line transfected at N/P 30 (r_s_ = 0.96; *p*<0.05). Of note, only copolymers with a degree of grafting between 5.2% and 8.8% were more cytotoxic than 2 kDa bPEI (*p*<0.05), with values ranging from 27.6±2.0% to 34.0±5.6%, whilst chitosan-based polyplexes were not toxic at all to HeLa cells (cytotoxicity: 0.3±2.7%), as previously shown also by others [34]. Unfortunately, a direct comparison between our results and the literature was not possible principally because previous data referred to the cytotoxicity of copolymers per se [13], [16], [21] instead of polyplexes at different N/P ratios as we did. According to most of existing studies we have evaluated the overall cytotoxicity which depends on the intrinsic toxicity of each component, on the biophysical properties of the corresponding polyplexes as well as on the dose of complexes administered to cells [35]. Indeed, Godbey and Aravindan showed that PEI does exert dual and distinct detrimental effects due to the free PEI in solution and to the more cytotoxic polyplex assemblies [28], [36], [37]. Moreover, it is worth noting that in our experimental conditions and at the highest N/P ratio tested (N/P 30), only 3.09 µg/mL of bPEI formulated in polyplexes were administered to cells, far below the concentrations (up to 1 mg/mL of free polymer) reported as highly cytotoxic by many other authors [7], [16], [17].

**Figure 5:**
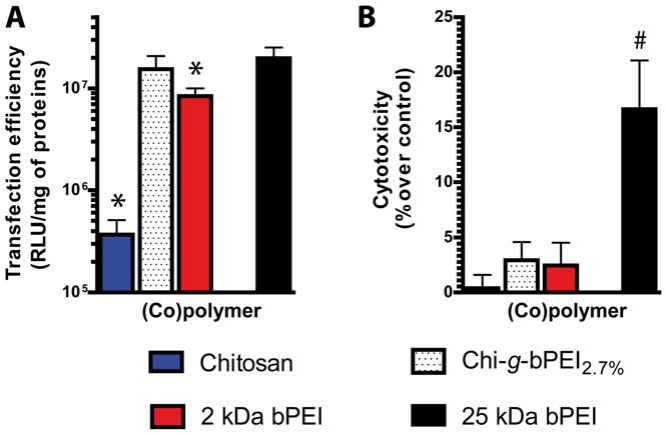
Transfection efficiencies and cytotoxicities of chitosan, Chi-*g*-bPEI_2.7%_ copolymer and bPEIs in primary cells. Transfection efficiencies (A) and cytotoxicities (B) in bovine articular chondrocytes. Chitosan, Chi-*g*-bPEI_2.7%_ copolymer and 2 kDa branched polyethylenimine (bPEI) were used at nitrogen (N) to plasmid DNA phosphate (P) ratio (N/P) 30 and 25 kDa bPEI was administered at N/P 10 according to the existing literature. Results are expressed as mean ± standard deviation (n≥5) (*, *p*<0.05 *vs.* Chi-*g*-bPEI_2.7%_; #, *p*<0.05 *vs.* 2 kDa bPEI).

It is generally believed that non-toxic conditions and/or gene delivery vectors are the prelude to poor transfection results [6], as we have previously reported [38]–[40] and as we are describing here for Chi-*g*-bPEI_0.6%_ and chitosan. Interestingly, along the copolymer series, Chi-*g*-bPEI_2.7%_ at N/P 30 was not very cytotoxic (19.7±3.3%) (Fig. 4A) but was the most effective in transfecting HeLa cells (4.4±1.4×10^7^ RLU/mg of proteins) (Fig. 4B), underscoring for the first time the fundamental role of the degree of grafting in modulating the overall transfection behavior of copolymers for gene delivery. On this ground, N/P 30 was chosen for a comparative evaluation of all Chi-*g*-PEI_x_ in two other cell lines (Fig. 4C–F). As expected, the transfection efficiency and the cytotoxicity of copolymers were dependent to a great extent on the cell type and, according to Boussif and colleagues [4], COS-7 were by far the most effectively transfected cells. Nevertheless, in two cell lines other than HeLa, the transfection profiles as a function of the degree of grafting of the Chi-*g*-PEI_x_ series followed a bell-shaped curve that gave the best value for Chi-*g*-bPEI_2.7%_ and the degree of grafting was correlated with the cytotoxicities of polyplexes (COS-7: r_s_ = 0.89; *p*<0.05 and U87-MG: r_s_ = 0.82; *p*<0.05, respectively). Interestingly, Chi-*g*-bPEI_2.7%_ was always more efficient in transfection than the starting materials chitosan and 2 kDa bPEI (*p*<0.05) and as cytotoxic as the LMW bPEI (not statistically significant). Although in our experimental conditions chitosan was really effective in complexing nucleic acids, the aminoglycoside gave a transfection efficiency around two order of magnitude lower than that of the gold standard 25 kDa bPEI, as previously reported also by Jiang and coworkers [13]. Since Chi-*g*-bPEI_2.7%_ at N/P 30 was very effective in transfecting relatively easy-to-transfect cell lines, we faced the problem of treating primary cells, focusing on chondrocytes that are known to respond poorly to non-viral vector-based approaches [41]. In light of this, when the aforementioned conditions were applied to transfect primary articular chondrocytes, both 25 kDa bPEI and Chi-*g*-bPEI_2.7%_ revealed very low transfection levels and were not cytotoxic at all (data not shown). Nevertheless, since the transgene expression is related to the plasmid dose administered to cells for a given N/P ratio [4] we decided to double the amount of polyplexes during transfection. In these conditions, Chi-*g*-bPEI_2.7%_ was as effective as 25 kDa bPEI in transfecting articular chondrocytes (15.5±5.4×10^6^ RLU/mg of proteins *vs.* 19.8±5.4×10^6^ RLU/mg of proteins, respectively; not statistically significant), but was significantly less cytotoxic (2.9±1.6% *vs.* 16.6±4.4%, respectively; *p*<0.05) (Fig. 5).

**Figure 6:**
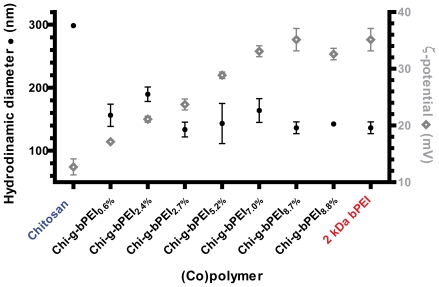
Physico-chemical characterization of chitosan-, copolymer- and 2 kDa bPEI-based polyplexes. Hydrodynamic mean diameter (full black circles) and ζ-potential (empty grey rhombi) of chitosan-, copolymer- and 2 kDa branched polyethylenimine (bPEI)-based polyplexes at nitrogen (N) to plasmid DNA phosphate (P) ratio (N/P) 30. Results are expressed as mean ± standard deviation (n≥4).

In light of transfection results and in order to evaluate the possible influence of the degree of grafting on the physico-chemical behavior of their assemblies with DNA, N/P 30 was chosen to compare the size and the ζ-potential of all Chi-*g*-PEI_x_-based polyplexes (Fig. 6). There is compelling evidence that the size and the surface charge of nanoparticles are important factors in modulating their cellular uptake [42]. Interestingly, the relatively homogeneous size distributions of all complexes were unimodal. Most important, since all complexes had almost the same hydrodynamic mean diameter (from 134±12 nm to 189±12 nm), there was no clear and significant trend between the size of polyplexes and the degree of grafting of copolymers, as depicted in Fig. 6. It is worth noting that these results are consistent with the general idea that small particle size is fundamental for attaining high transfection efficiency [43]. Inversely, although for a given N/P ratio (e.g. N/P 30) the bPEI content for each formulation was the same, the ζ-potential of Chi-*g*-bPEI_x_-based polyplexes increased with increasing the degree of grafting of copolymers (from +17±1 mV to +35±2 mV). Taken together, the higher the degree of grafting, the greater the ζ-potential of the resulting polyplexes (r_s_ = 0.96; *p*<0.05) and their cytotoxicity in all cell lines tested (Fig. 4B, 4D and 4F).

In conclusion, the development of new copolymers for gene delivery has long been an iterative process: copolymers have been designed one at a time and individually tested for their physico-chemical and biological properties [13], [14], [16], [21], [22]. In this work we have synthesized and characterized a series of seven Chi-*g*-bPEI_x_ copolymers with a variable amount of bPEI grafted onto chitosan in order to provide a first, comprehensive structure-activity relationship study. Indeed, this work underlines for the first time the pivotal role of the degree of grafting on the overall transfection behavior of copolymers. We have demonstrated that subtle changes in the degree of grafting along the copolymer series directly affected the overall charge of polyplexes and, altogether, had a direct effect on cytotoxicity. Of note, along the Chi-*g*-bPEI_x_ series, an intermediate degree of grafting of 2.7% conferred the highest transfection efficiency and low cytotoxicity in three cell lines transfected in complete medium. Finally, in transfection experiments carried out with primary articular chondrocytes, Chi-*g*-bPEI_2.7%_ was as effective as the gold standard 25 kDa bPEI and it showed little cytotoxicity. Nevertheless, further research is required to find out possible explanations for the correlation between the chemical structure of Chi-*g*-bPEI_x_ and the biological properties of the corresponding complexes with DNA. In this scenario, ongoing studies aim to shed light on putative mechanisms of DNA complexation with different copolymers that might explain our findings.

## Materials and Methods

### Materials

Plasmid DNA encoding for the modified firefly luciferase pGL3-Control Vector (5.2 kb) and Luciferase Assay System were purchased from Promega (Milan, Italy), anion-exchange columns for purification of plasmid DNA were obtained from Qiagen (Milan, Italy) and BCA Protein Assay Kit was from Pierce Chemical (Rockford, IL, USA). HeLa (human cervix carcinoma, CCL-2.2), U87-MG (human glioblastoma-astrocytoma epithelial-like, HTB-14) and COS-7 (African green monkey kidney fibroblast-like, CRL-1651) cell lines were purchased from the American Type Culture Collection (ATCC, Manassas, VA, USA) while *E. coli* DH5α (Cat. 9027) was from Takara Bio (Otsu, Japan). MMW chitosan (MW 1.9–3.1×10^5^; deacetylation degree: 75–85%; Brookfield viscosity: 200–800 cP, 1% in 1% CH_3_COOH), 2 kDa bPEI (Mn∼1.8×10^3^, MW∼2.0×10^3^), 25 kDa bPEI (Mn∼1.0×10^4^, MW∼2.5×10^4^) and all other chemicals were from Sigma-Aldrich (Milan, Italy) if not differently specified. Use of a cationic polymer based on PEI for transfection is covered by US Patent 6,013,240, European Patent 0,770,140, and foreign equivalents, for which Polyplus-transfection™ is the worldwide exclusive licensee. ^1^H NMR spectra were run on Bruker spectrometer 400 MHz. Chemical shifts are expressed in ppm (δ), using tetramethylsilane (TMS) as internal standard for ^1^H nucleus (δ_H_ = 0.00). Fluorimetric and spectrophotometric analysis were performed using GENios Plus reader (Tecan, Segrate, Italy).

### Synthesis of Chi-g-bPEIx copolymers

Chi-*g*-bPEI_x_ copolymers at different degrees of grafting of 2 kDa bPEI onto the chitosan backbone were synthesized with minor modifications of the two-step procedure previously reported by Jiang and colleagues [13]–[16], [21], [22] involving the imine reaction between the primary amino groups of bPEI and periodate-oxidized chitosan, as depicted in Fig. 1. The degree of grafting was represented as the index “x” that is the percentage of GlcN monomers bearing 2 kDa bPEI grafts. Briefly, the oxidation step was performed by treatment of MMW chitosan with a slight excess of KIO_4_ in sodium acetate buffer (pH 4.4), previously degassed with N_2_ bubbling and kept at 4°C. The reaction was run for 48 h at room temperature (r.t.) and quenched by adding 10% v/v ethylene glycol. The resulting periodate-oxidized chitosan was purified following a two-step dialysis procedure (Spectra/Por membrane: MWCO = 1.0×10^4^) first against 0.2 M NaCl (pH 4.5) and finally against milli-Q water (pH 4.5) and was then treated with 1.27×10^−2^, 6.35×10^−2^, 1.27×10^−1^, 6.35×10^−1^, 2.54×10^0^, 6.35×10^0^, and 1.27×10^1^ equivalents of 2 kDa bPEI, calculated with respect to the number of deacetylated units (GlcN) within the oxidized chitosan backbone. Each mixture was stirred for 48 h at 4°C, before the addition of NaBH_4_. The resulting solution was dialyzed as above and finally freeze-dried.

### Characterization of copolymers

The compositions of the intermediates and of Chi-*g*-bPEI_x_ copolymers were evaluated by ^1^H NMR. The amine content and the degree of grafting of bPEI onto the chitosan were determined by an amine titration procedure, namely the 2,4,6-trinitrobenzene sulfonic acid (TNBSA) assay [44]. Briefly, chitosan, Chi-*g*-bPEI_x_ and bPEIs were diluted to 20 mg/mL in 0.1 M NaHCO_3_ (pH 8.5). TNBSA was added to each sample to a final concentration of 0.0033% and the resulting solutions were incubated for 2 h at 37°C. A standard curve of pentylamine was used for the determination of primary amines on polymers by measuring the absorbance of each sample at 335 nm. The degree of grafting was expressed as percentage of average chitosan GlcN units grafted with bPEI:
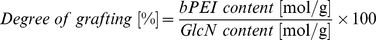



### Preparation of plasmid DNA

pGL3-Control Vector plasmid was transformed in *E. coli* DH5α and amplified in LB broth media at 37°C overnight. The plasmid was purified with maxiprep anion-exchange columns according to the manufacturer's protocol. The purity of plasmid DNA was assessed by the absorbance ratio at OD_260_/OD_280_. Plasmid DNA was stored at −20°C until use.

### Preparation of polyplexes

Chitosan was dissolved in 0.1% CH_3_COOH in water and diluted in 10 mM Hepes buffer (pH 7.0). Polyplexes were prepared at r.t. by adding an aqueous solution of pGL3 to aqueous solutions of chitosan, copolymers or bPEI in 10 mM Hepes buffer (pH 7.0), at the desired polymer concentration, yielding different N/P ratios. The resulting mixtures were further incubated for 30 min at r.t.

### Fluorophore-exclusion assay

The nucleic acid binding ability of chitosan, copolymers and bPEIs was monitored by a fluorophore-exclusion assay [45], [46]. Briefly, polyplexes at different N/P ratios were prepared complexing 40 ng of pGL3 in a final volume of 20 µL and incubated for 30 min at r.t., as described above. Then, complexes were diluted 1∶5 in 10 mM Hepes buffer (pH 7.0) containing 0.67 µL of SYBR Green I 200× (λ_ex_ = 497 nm; λ_em_ = 520 nm) and incubated for 15 min at r.t.. The fluorescence of the intercalated dye was measured in black 96-well microplates. The relative fluorescence (*F*) was determined as follows:
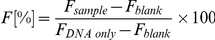



### Gel retardation assay

The DNA complexation ability of copolymers was further assessed by gel retardation assay on a 0.75% agarose gel [39]. Complexes were prepared at the desired N/P ratio, as described above. Samples (300 ng of pGL3 in a final volume of 15 µL) were added to 3 µL of loading dye (0.05% bromophenol blue, 40% sucrose, 0.1 M ethylenediaminetetraacetic acid (EDTA, pH 8.0) and 0.5% sodium dodecyl sulfate) and electrophoresed at 100 V for 1 h in Tris-Acetate-EDTA (TAE) buffer. The DNA bands were visualized with Kodak Image Station 440 CF (Kodak, Milan, Italy) after incubating the gel with SYBR Green I in TAE buffer.

### Measurement of size and ζ-potential of polyplexes

Dynamic Light Scattering (DLS) studies were performed using a Malvern Zetasizer Nano ZS instrument (Malvern Instruments, Worcestershire, UK), fitted with a 633 nm laser at a fixed scattering angle of 173°. The ζ-potential of polyplexes was measured by Laser Doppler Velocimetry in the same apparatus, measuring the electrophoretic mobility with Phase Analysis Light Scattering (PALS) technique. For each condition, 1 µg of pGL3 was added to 46 µL of chitosan, Chi-*g*-bPEI_x_ or bPEI solutions at different concentrations in order to achieve the desired N/P ratio. The resulting mixtures were incubated for 30 min at 25°C, further diluted 1∶9 in 10 mM Hepes buffer (pH 7.0) and equilibrated at 25°C prior to measurements.

### Cell culture and transfection

Articular chondrocytes were isolated from metacarpophalangeal joints of 8-month-old calves, according to the previously described procedure [47]. Primary cells and cell lines were cultured at 37°C in a humidified atmosphere of 5% CO_2_ in Dulbecco's Modified Eagle Medium (DMEM) supplemented with 1 mM sodium pyruvate, 10 mM Hepes buffer, 100 U/mL penicillin, 0.1 mg/mL streptomycin, 2 mM glutamine, and 10% FBS (complete DMEM). For transfection experiments, cells were plated in 96-well cell culture plates at a density of 1.5×10^4^ cells/cm^2^. Twenty-four hours after cell seeding, 51.2 ng/well of pGL3 were complexed as previously described with chitosan, Chi-*g*-bPEI_x_ or bPEIs at the desired N/P ratio and then added to cells in a final volume of 64 µL/well of complete DMEM. According to the existing literature, 25 kDa bPEI was used at N/P 10 [16], [20]. Twenty-four hours post transfection, polyplex cytotoxicity was assessed using AlamarBlue cell viability assay from Life Technologies Italia (Monza, Italy) according to manufacturer's guidelines. Viability of untreated control cells was assigned as 100% and cytotoxicity was determined as follows:
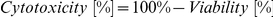



Finally, cells were lysed with Cell Culture Lysis Reagent (Promega Italia, Milan, Italy) and luciferase activity was measured by Luciferase Assay System using a Mithras luminometer (Berthold Technologies, Brugherio, Italy) and normalized to the cellular protein content, evaluated by BCA assay.

### Statistical analysis

Statistical analysis was carried out by GraphPad version 5.04 (GraphPad software, La Jolla, CA, USA). Comparisons among groups were performed by one-way ANOVA, and Tukey's multiple comparison test post hoc. Correlations between variables were calculated using Spearman's correlation coefficient test and *p* values were two-sided. Significance was retained when *p*<0.05. Data are expressed as mean ± standard deviation (SD).

## Supporting Information

Figure S1 **Fluorochrome exclusion assay of (co)polymers as a function of N/P ratio.** Comparative evaluation of DNA complexation abilities of chitosan (A) and Chi-*g*-bPEI_x_ copolymers (B-H) (black squares and lines) with respect to 2 kDa branched polyethylenimine (bPEI) (red stars and lines) evaluated by monitoring the SYBR Green I-fluorochrome exclusion from polyplexes as a function of nitrogen (N) to plasmid DNA phosphate (P) ratio (N/P). Results are expressed as mean (*n*≥3).
(TIF)

Figure S2 **Agarose gel electrophoresis of Chi-** ***g*** **-bPEI_x_/plasmid DNA polyplexes as a function of N/P ratio.** Gel retardation (shift) assays of Chi-*g*-bPEI_x_ copolymers as a function of nitrogen (N) to plasmid DNA phosphate (P) ratio (N/P).
(TIF)

Figure S3 **Transfection efficiencies and cytotoxicities of (co)polymers at increasing N/P ratio.** Transfection efficiencies (A) and cytotoxicities (B) of (co)polymers in HeLa cell line. Chitosan, Chi-*g*-bPEI_x_ copolymers and 2 kDa branched polyethylenimine (bPEI) were used at increasing nitrogen (N) to plasmid DNA phosphate (P) ratios (N/P) and 25 kDa bPEI was administered at N/P 10 according to the existing literature. Results are expressed as mean ± standard deviation (*n*≥4) (*, *p*<0.05 *vs.* Chi-g-bPEI_2.7%_ for a given N/P ratio. #, *p*<0.05 *vs.* 2 kDa bPEI for a given N/P ratio).
(TIF)
