# Supplementary figures and images for: Chitosan-Graft-Branched Polyethylenimine Copolymers: Influence of Degree of Grafting on Transfection Behavior
Source: PLoS One. 2012 Apr 11;7(4):e34711. doi: 10.1371/journal.pone.0034711 (PMC3324502; doi:10.1371/journal.pone.0034711)

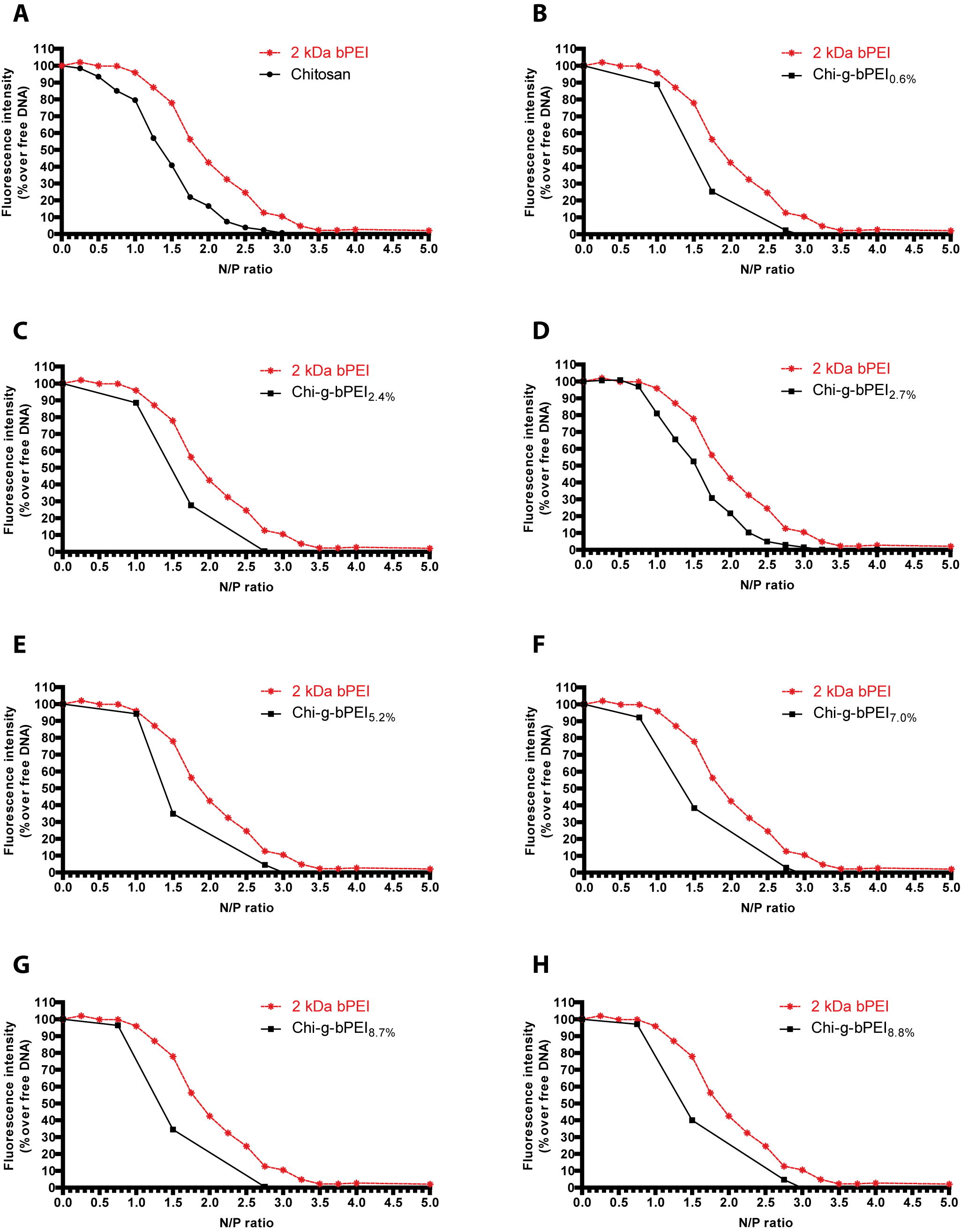

Supplement: Figure S1 — Fluorochrome exclusion assay of (co)polymers as a function of N/P ratio. Comparative evaluation of DNA complexation abilities of chitosan (A) and Chi-g-bPEIx copolymers (B-H) (black squares and lines) with respect to 2 kDa branched polyethylenimine (bPEI) (red stars and lines) evaluated by monitoring the SYBR Green I-fluorochrome exclusion from polyplexes as a function of nitrogen (N) to plasmid DNA phosphate (P) ratio (N/P). Results are expressed as mean (n≥3). (TIF) [file pone.0034711.s001.tif]

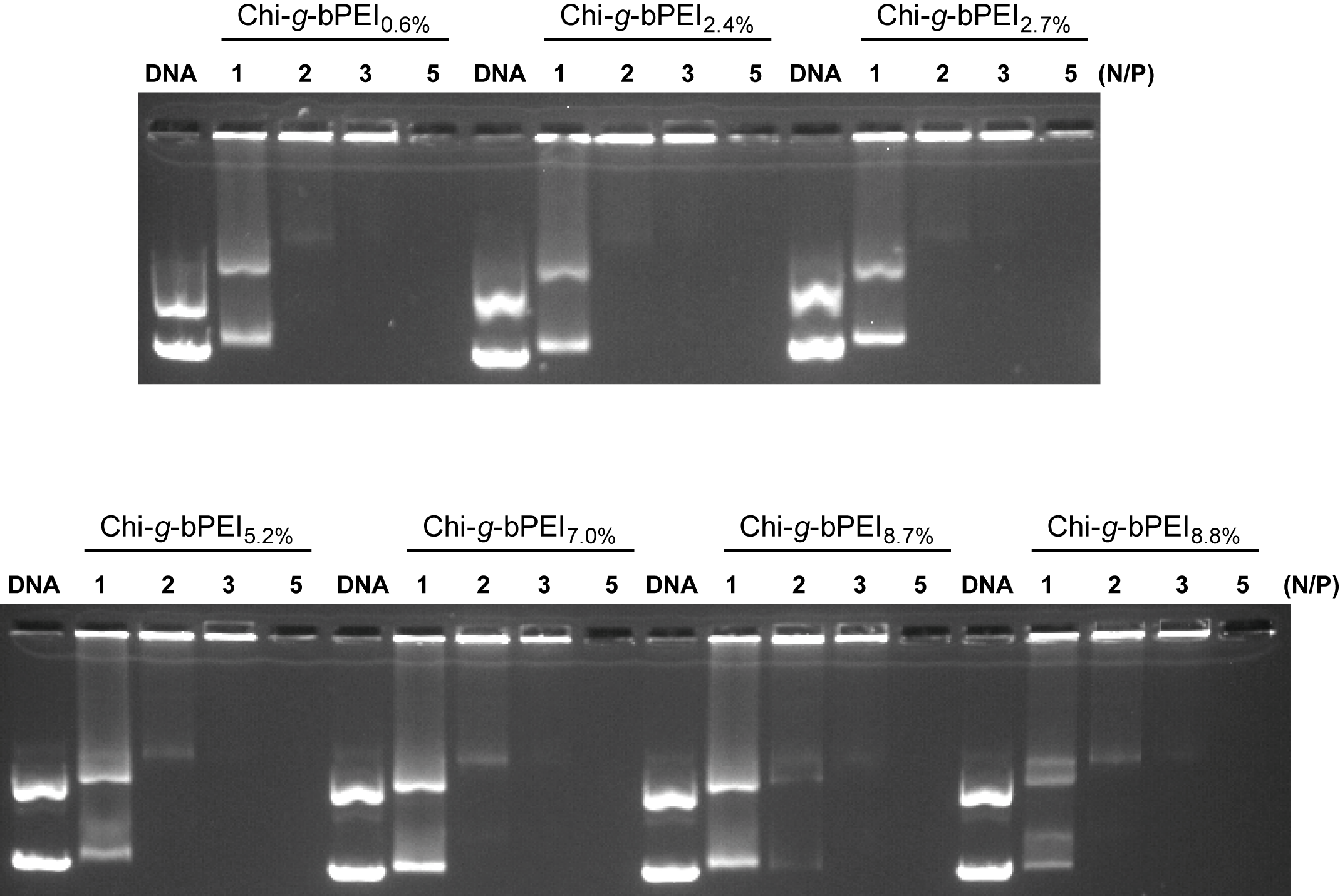

Supplement: Figure S2 — Agarose gel electrophoresis of Chi- g -bPEIx/plasmid DNA polyplexes as a function of N/P ratio. Gel retardation (shift) assays of Chi-g-bPEIx copolymers as a function of nitrogen (N) to plasmid DNA phosphate (P) ratio (N/P). (TIF) [file pone.0034711.s002.tif]

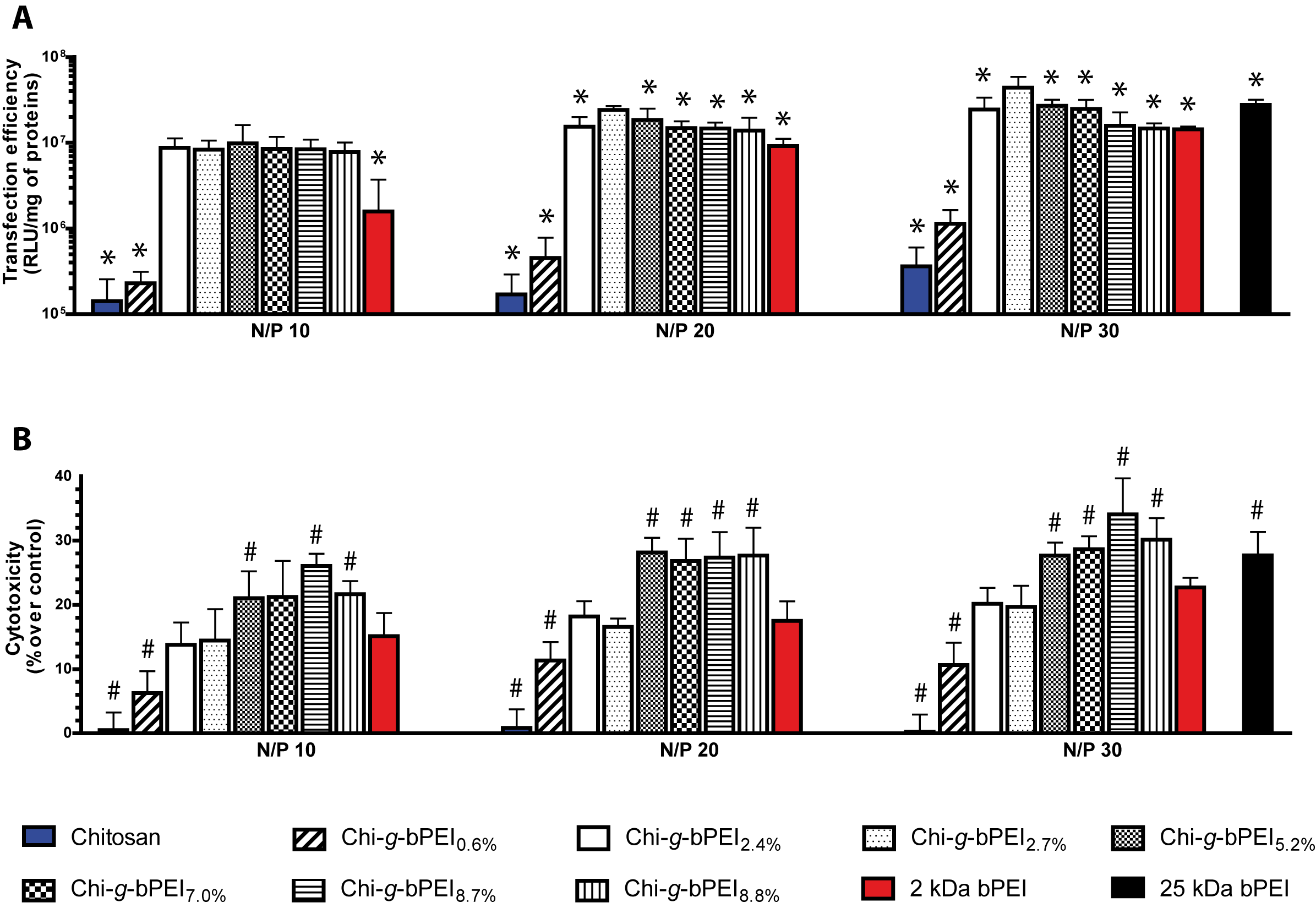

Supplement: Figure S3 — Transfection efficiencies and cytotoxicities of (co)polymers at increasing N/P ratio. Transfection efficiencies (A) and cytotoxicities (B) of (co)polymers in HeLa cell line. Chitosan, Chi-g-bPEIx copolymers and 2 kDa branched polyethylenimine (bPEI) were used at increasing nitrogen (N) to plasmid DNA phosphate (P) ratios (N/P) and 25 kDa bPEI was administered at N/P 10 according to the existing literature. Results are expressed as mean ± standard deviation (n≥4) (*, p<0.05 vs. Chi-g-bPEI2.7% for a given N/P ratio. #, p<0.05 vs. 2 kDa bPEI for a given N/P ratio). (TIF) [file pone.0034711.s003.tif]
